# The global surgery blood drought: frontline provider data on barriers and solutions in Bihar, India

**DOI:** 10.1080/16549716.2019.1599541

**Published:** 2019-04-25

**Authors:** Rachita Sood, Rachel R Yorlets, Nakul P Raykar, Remya Menon, Hemant Shah, Nobhojit Roy

**Affiliations:** a Division of Plastic and Reconstructive Surgery, Northwestern University, Chicago, IL, USA; b Program in Global Surgery and Social Change, Harvard Medical School, Boston, MA, USA; c Department of Plastic and Oral Surgery, Boston Children’s Hospital, Boston, MA, USA; d Department of Surgery, Beth Israel Deaconess Medical Center, Boston, MA, USA; e CARE India – Bihar, Patna, Bihar, India; f The Program in Global Surgery and Social Change, Department of Public Health Sciences, Health Systems and Policy, Karolinska Institutet, Stockholm, Sweden

**Keywords:** Blood transfusion system, maternal mortality, India, health system strengthening, obstetric hemorrhage, global surgery

## Abstract

**Background**: Limited access to safe, timely banked blood is a critical barrier to providing basic surgical care in resource-limited settings globally. Contextual, locally driven data are required to elucidate country needs, develop effective interventions, and guide policy decisions.

**Objective**: We employ qualitative methodology to describe barriers faced and solutions proposed by front-line obstetric providers in Bihar – a poor, populous Indian state where maternal mortality exceeds the national average. We aim to make locally driven recommendations for ongoing policy work in India to strengthen the country’s blood transfusion system.

**Methods**: From February to May 2016, two researchers conducted semi-structured interviews with 19 obstetric providers across Bihar. Snowball sampling was employed until thematic saturation was reached. Following immersion into de-identified texts and dual codebook development, a primary analyst completed topical coding, and a secondary analyst confirmed reproducibility.

**Results**: Providers report that pervasive banked blood shortages force hospitals to require replacement donation, but patients’ families often cannot or will not donate. Providers wait one to six hours for blood, depending on availability of staff and supplies, blood bank proximity, and the ability of the patient being treated to navigate the system. Providers feel forced to refer their patients, often to distant, poorly equipped centers. Providers identify donor education, improved infrastructure, and improved local coordination as focus areas for intervention.

**Conclusions**: A multi-stakeholder approach that aims to increase blood donation through community education, mitigate limited infrastructure through short-term workarounds, and improve local-level coordination through state support and policy change is required in Bihar. This study generates data to guide policy and future research aimed at generating affordable, contextually appropriate interventions to the blood drought.

## Background

Limited access to safe, timely banked blood is a critical barrier to providing basic surgical care – including cesarean and operative delivery – in resource-limited settings globally [1–3]. Indeed, an estimated 150,000 pregnancy-related deaths could be averted yearly through access to safe blood for operative and non-operative management of hemorrhage [4,5]. The Lancet Commission on Global Surgery (LCoGS) calls for global access to a safe and affordable blood supply by 2030 through a minimum collection of 15 units per 1000 people/year; low- and middle-income countries (LMIC) collect a median 3.9–11.7 units per 1000 people/year [1,6]. To overcome this deficit, LMIC surgeons describe heroic workarounds, including donating their own blood or using an unbanked, direct blood transfusion (UDBT) from a willing donor, even in a context where the practice is illegal and may lead to provider imprisonment [7,8]. These drastic measures are often not enough to save the patient.

**Figure 1.**
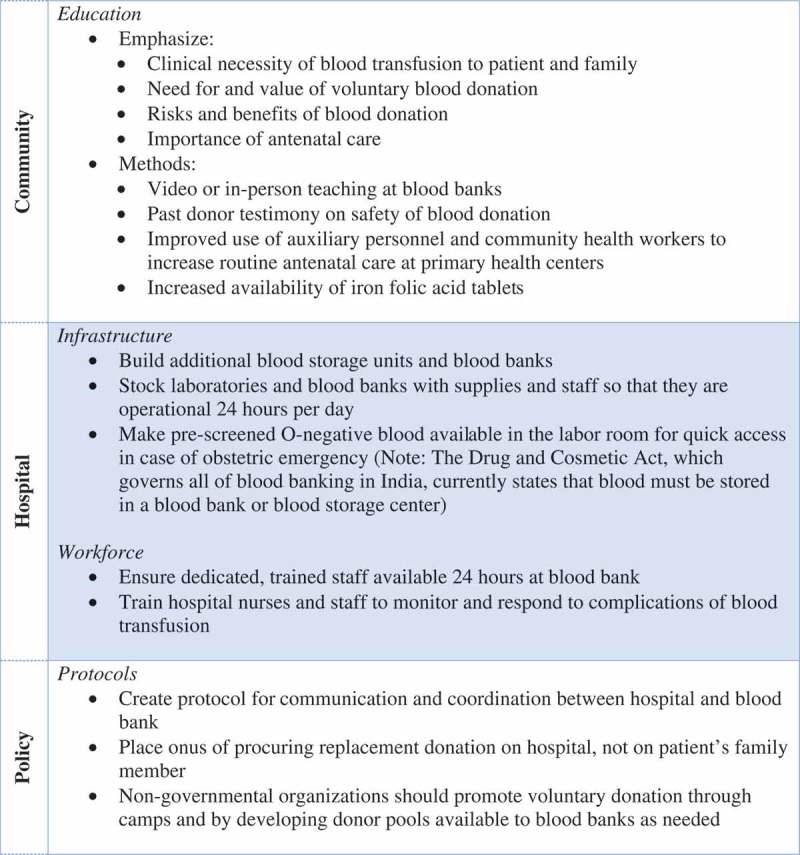
Interventions proposed by obstetric care providers to improve their ability to care for women who require blood transfusion.

Surgeons’ access to blood is especially relevant in India, where less than 3% of the population is estimated to have timely, affordable access to surgical care and one-fifth of the world’s maternal deaths occur [9,10]. As in many LMIC, in India an insufficient base of voluntary, non-remunerated donors, limited workforce and infrastructure, and a poorly coordinated blood transfusion system limit access to blood [11,12]. We aim to further characterize context-specific barriers and describe novel solutions to the surgical blood drought in India through in-depth interviews with obstetric care providers in the Indian state of Bihar. Bihar – India’s third most populous (104.1 million people in 2011) and poorest state by gross domestic state product (GDSP) per capita [13,14]. Rigorous qualitative data from the experiences of frontline providers in this context are critical to inform global surgery implementation and research agendas to meet the LCoGS 2030 goal.

## Methods

We conducted a cross-sectional, qualitative study consisting of in-depth interviews with 19 obstetric care providers across the state of Bihar. A grounded theory approach was used to analyze interview data and determine key themes regarding providers’ ability to access blood and proposed solutions. We adhered to the consolidated criteria for reporting qualitative research (COREQ) [15].

### Participant selection

Inclusion criteria were physicians (1) with an MBBS – the undergraduate medical degree in India – or additional training in obstetrics and gynecology (e.g. CEmOC training, a diploma course, or an MD or MS) (2) who currently practiced obstetrics in private, public, or non-governmental organization (NGO) settings across the state of Bihar. From February to 15 May 2016, two female interviewers (RS, RM) conducted snowball sampling, by phone and in person, to recruit providers until thematic saturation was reached [16]. At the time of data collection, RS was a USA-based medical student on a nine-month research grant in Patna, Bihar and RM was an Indian physician (MBBS DGO) employed by a Patna-based non-governmental organization.

### Data collection

A semi-structured interview tool was created to explore providers’ need for blood transfusion, challenges to meeting that need, and key interventions that obstetric care providers would implement, if possible [17]. The structure of questions was based on an interview guide used previously to interview 148 surgical care providers in low-resource settings in 21 countries [7]. An interview implementation manual was developed to standardize interview protocol between the two interviewers.

Both interviewers are native speakers of Hindi and fluent in English, the two languages in which interviews were conducted. Interviewees knew the interviewers’ backgrounds. Participants were informed that interviews would be de-identified, uncompensated, and compiled into a research study. Interviews were completed in person (14 interviews) – in private spaces in the obstetric care providers’ places of work – or over the phone (5 interviews). Interview duration ranged from 20 to 60 minutes. Interviewers used probes and unprompted follow-up questions [18]. Field notes were recorded during or after interviews in English, with the exception of several quotes, which were translated into English by native speakers. All texts were de-identified. Field notes were not returned to participants for comment.

### Data analysis

Following immersion into field notes, two authors (NPR, RY) identified key topics and themes from the first five interviews using grounded theory to develop a coding manual for analyzing future interviews [19]. The primary analyst (RY) completed coding, adapting and expanding the coding manual as appropriate, and confirming reproducibility of codes with the second analyst as necessary. Data were coded topically in Microsoft Word and organized into respective codes in Microsoft Excel. Final themes were summarized into an outline, which was returned to the interviewers for review and feedback.

## Results

Two research team members interviewed 19 obstetric care providers with varying credentials and practice settings (Table 1). The direct quotes and field excerpts included below are illustrative of emergent themes (Table 2).

**Table 1.** Characteristics of interviewed providers.

Providers’ gender, n (%)										
	Female									17 (89%)
	Male									2 (11%)
Highest degree obtained by provider, n (%)*										
	MD or MS									9 (47%)
	Diploma course									3 (16%)
	CEmOC Training									4 (21%)
	MBBS									3 (16%)
Providers’ practice setting, n (%)º										
	Tertiary care center									5 (20%)
	District hospital									11 (44%)
	Private facility									8 (32%)
	Charitable facility									1 (4%)

*Diploma and MD or MS are two different post-graduate pathways for MBBS trained doctors to sub-specialize in obstetrics and gynecology. CEmOC is ‘Comprehensive Emergency Obstetric Care,’ a 16-week training course for MBBS-trained physicians in emergency obstetric care, including cesarean section and management of obstetric complications [47]. MBBS is the undergraduate medical degree in India.

ºWe defined a tertiary care center as a medical college, a district hospital as a hospital designated as such by the state government of Bihar, a private facility as a practice run outside of state government infrastructure, and a charitable facility as one managed by a non-governmental organization [47]. Note – six providers reported working in more than one of these types of facilities.

**Table 2.** Key themes identified in provider interviews.

Themes	Illustrative Quotes and Excerpts
**Theme 1**: Pervasive blood shortages force facilities to require replacement donation	‘That is why this whole problem of donor exchange is happening. [Blood banks] have no option but to ask for something in return. At some points they would have just 20 bags. So if you keep giving blood, your blood will be gone in a day’ [Participant number 3].
Blood availability is variable	When there are severe anemia cases like who come to us with hemoglobin 6 then it is important to transfuse on time. At that time, the blood bank doesn’t have it. We also can’t get a donor. So blood is not available [Participant number 9]. Blood availability is no longer an issue at our facility due to help from our support partners. We also have a tie-up with red cross, which provides our patients with blood when our blood bank is short of units, especially negative blood [Participant number 16].
Patient education and cultural norms	‘There is havoc among people that, I’ve given blood, I’m going to die… Twenty attendants will be there and then run away. …The lady [patient] herself will say, “Don’t call my husband, don’t call my son”’ [Participant number 4].
**Theme 2**: When blood is available, additional barriers delay or prevent transfusion	‘If we ask for blood in the morning, then we will get it by evening. It easily takes six hours. For educated patients, it can even take two or three hours. But the patients who are illiterate have a lot of problems’ [Participant number 9].
Geographic barriers	We usually don’t ask for blood transfusion prior to transfer as our blood bank is 30–45 minutes away (in old district hospital campus) and getting blood can be time consuming, which can further agitate the relatives [Participant number 14].
Infrastructure	So the first thing would be that there would be no bulbs – to collect blood from patient and send it to BB for cross matching [Participant number 3]. Our hospital is not well equipped to handle any blood transfusion reaction [Participant number 18]. No facilities for investigating patients’ hemoglobin levels, so that we can convince relatives for the need for blood transfusion. Also most patients are not registered and have no idea about their blood group and as we don’t have a 24-hour lab we cannot determine their blood group [Participant number 18].
Workforce	There is not adequate staff at the blood bank so there is a delay in the process [Participant number 6]. Our nurses are unaware of the blood transfusion protocols and are unwilling to transfuse blood during their shift fearing transfusion reactions. No blood transfusions are done after 5 pm at our facility [Participant number 19].
Protocols for coordination	‘There was no liaison between the hospital and the blood bank… no protocol in place to get blood quickly. Even if you send the relative to go to Red Cross blood bank he would be lost. So many times we would be waiting one and a half hours – where is the relative? There would be no one to show him the way to the blood bank. These people are coming from rural areas – they would get lost’ [Participant number 3].
Affordability of blood	‘The most important is cost. It costs 2000–2500 INR [30 to 38 USD] if replacement donation is not given. If replacement is given, then it costs 800 INR.’ The poor patients who come, for them the cost is too much and their hemoglobin is low. The educated patients who come. Their hemoglobin is good and the cost is also okay. Educated and poor patients come to my clinic. Both sometimes refuse blood transfusion [Participant number 11].
**Theme 3**: Disparities exist between public and private care settings	In private practice we have to own everything we do. Like if we don’t treat the patient well… in the district hospital there isn’t good treatment given [Participant number 11].
**Theme 4**: Providers work around blood shortages	‘Sometimes there is a delay so we give colloids to keep the blood pressure stable.’ Sometimes we give injectable iron. For elective cases: ‘On the operation day, the donor stays in [the medical college hospital] near the blood bank. If blood is required, we get it and there is no delay’ [Participant number 6].
Providers acquire blood without replacement in emergencies	If they are too poor to get blood, we have nowhere to send them. The post-graduate goes to the blood bank and writes ‘MND’ – money and donor not available. Every day there is one patient like this [Participant number 8].
Mutual vulnerability forces referral	For example: an unregistered multiparous woman was brought in an auto-rickshaw to our district hospital with antepartum hemorrhage and before the relatives could arrange for blood as ordered by the on duty doctor, the patient died. There was resultant mob violence and since then we have decided to refer all such complicated patients to higher center and not take any risks [Participant number 14].
**Theme 5**: Blood shortages affect patient care	A lot of times diagnosed case of placenta previa or uterine rupture… these cases would be referred to the district hospital and the surgeon would refuse to touch the patient without blood. And when you don’t get blood or you get blood within 3–4 hours either the patient is already dead or the doctor has already left [Patient number 3].

### Theme 1: pervasive blood shortages force facilities to require replacement donation

Providers report pervasive shortages of blood. In a tertiary care center, a district hospital, and three private facilities, providers note a particular shortage of type A and AB negative blood [Participants 1, 4, 6, 7, 9]. One district hospital provider states ‘I have not used blood in any case in the last six months. It was not like I didn’t need it. I didn’t get it’ [Participant 3]. However, the shortage is not universal. A district hospital physician states ‘Blood availability is no longer an issue at our facility due to help from our support partners. We also have a tie-up with red cross, which provides our patients with blood when our blood bank is short of units, especially negative blood’ [Participant 16].

Limited availability forces blood banks to require replacement donation – one unit of blood to the blood bank in exchange for each unit given to the patient. Providers reported that patients’ families often cannot (e.g. are anemic, unable to afford long trip to hospital) or will not provide a replacement donation. Providers believe that limited education on blood transfusion contributes to the latter – ‘The main reason for inadequate blood transfusions in our facility is that the patient’s [family members] are not aware of the need and the difference blood transfusions can make in management of a hemorrhaging patient’ [Participant number 5]. Additionally, providers report being told by family members that they fear becoming weak, unable to work, or dying from donating blood. Last, multiple providers reported that willingness to donate varies by sex; one provider stated, ‘Mothers are also very protective of their sons. They value sons over wives. They think that it is a very serious condition. If I give blood, I will be weak for life. It’s easier to get another wife’ [Participant number 8].

### Theme 2: when blood is available, additional barriers delay or prevent transfusion

Blood shortages and difficulty in acquiring replacement donation are compounded by geographic, infrastructural, and workforce barriers. Some blood banks are far from the hospital – a district hospital physician states, ‘Our blood bank is at the old district hospital campus approximately 30 to 45 minutes away’ [Participant 14]. Three participants interviewed at a three-year-old medical college without a blood bank state, ‘It takes a minimum of two hours to get blood from [the other state medical college]’ [Participant 6]. When the blood bank or storage unit is on the hospital campus, lack of coordination protocols and infrastructure may delay acquisition. In a tertiary care facility, a provider states, ‘The biggest problem is for that relative to reach the blood bank. If the hospital management takes the ownership that the moment blood is needed, this one person is made responsible’ [Participant 3]. When the blood bank or storage unit is off campus, providers cite poor road infrastructure and train timetables as delaying factors. In summary, providers estimate that they wait one to six hours to receive blood, depending the blood bank’s proximity and stock, the patient’s donor’s ability to navigate the system, and the time of day, because blood bank staff is limited at night.

Once the blood reaches the hospital, providers are without 24-hour facilities and personnel to determine hemoglobin level and blood type, essential supplies for the blood bank to cross-match and transfuse blood, adequately trained nurses to monitor the transfusion, or an intensive care unit for post-transfusion complications (Table 2, Infrastructure). One district hospital provider described the impact on patient safety, ‘Nobody would notice that they are going back with severe anemia. Because there would be no laboratory facilities available. The initial hemoglobin is not tested. The discharge hemoglobin is not tested. The patient is just alive’ [Participant number 3].

### Theme 3: disparities exist between public and private care settings

Providers also acknowledged disparity in accessing blood and surmounting the above barriers between their private clinic and public hospital practices. One described a patient with uterine rupture who presented with hemoglobin of 6 g/dL and survived, stating, ‘We got two pouches [of blood] at a time… If this case happened in the district hospital, then we would get beat. We would get fewer pouches [of blood], it would take longer time, and the patient would say, “What did you do, madam?”’ [Participant number 9].

Disparities across facilities can delay emergency cases. Patients at public facilities can only get blood from a public blood bank, even if a private blood bank is closer. This policy intends to prevent conflict of interest for government employees and protect patients from private blood banks’ sub-standard regulation. One district hospital physician stated, ‘And we say that we will not take the risk of the private. We don’t have permission that we tell the patient that you go and bring blood from the private… It should be that in emergencies the patient is allowed to get blood from outside. The patient is dying anyway, so taking a risk should be allowed’ [Participant number 9].

### Theme 3: providers work around blood shortages

When blood is delayed or not available, providers alter clinical management. Acutely, obstetricians give isotonic fluids to sustain hemorrhaging patients, injectable iron-sucrose to boost hemoglobin levels, and increased oxytocin intrapartum to prevent postpartum hemorrhage. In elective cases, providers maximize hemoglobin through transfusion weeks before surgery and send family members to the blood bank at the time of cesarean delivery. Private physicians described being able to perform such measures more commonly than district hospital physicians – ‘Private we also have difficulties but less because I am very particular. The day she comes for her ante-natal I write down family names and blood types. I also always have two of the patients’ attendants on delivery day’ [Participant number 4]. Additional workarounds include encouraging replacement donation by sending hospital staff to educate the patient or to ask obstructive relatives to leave. In emergencies, providers may ask blood bank staff for blood without replacement through administrative mechanisms.

### Theme 5: blood shortages affect patient care

The above-described barriers to obtaining blood alter patient management. For example, one provider states ‘Earlier we had 24 × 7 functional lab and we tested every patient for hemoglobin on arrival and all women with hemoglobin less than 7 were referred to [the medical college hospital] even before the active phase of labor. Since a few years, the lab is not functional after 2 pm’ [Participant 14]. In certain cases, providers feel forced to refer patients in unsafe conditions. In some cases, mutual vulnerability between patients who do not trust the providers and providers who fear violent backlash motivates referrals, as one provider described – ‘Imagine that hemoglobin is 3. Then you can’t do an operation, no matter how much of an emergency it is. That time we refer. We don’t take the patient because our situation here is that the patient will not understand you. If there is an on-table death, then they will say that you didn’t tell us. They won’t listen to anything. They will beat us. We know that the patient will die [when we refer], but we have no choice and have to look out for us. We can’t get blood immediately’ [Participant number 9].

### Theme 6: providers advocate for community, facility, and policy-level solutions

Participants advocated for changes at the community, facility, and larger policy levels to improve access to blood (Figure 1). At the community level, providers describe a need for education about the need for blood donation, the safety of blood transfusion, and the importance of antenatal care for prevention of anemia. A district hospital physician states ‘Relatives of high-risk women must be counseled to nominate a person for blood donation in case their patient requires blood transfusion at any stage of pregnancy or labor’ [Participant number 14]. A number of specific methods of education are described: video teaching at blood banks, a dedicated counselor at blood banks, and past donor testimony at donation camps regarding the safety of blood donation.

At the facility level, participants advocate for building additional blood banks and storage units. A district hospital provider states ‘the blood bank must be shifted to our new district hospital campus’ [Participant 14]; another district hospital participant states ‘there should be more blood banks in the periphery’ [Participant 8]. Existing blood banks and storage units, as well as hospitals, need to be stocked with adequate supplies to administer a blood transfusion. Finally, providers acknowledge a need for trained, 24 hour staff in blood banks and nurses in hospitals to administer blood and monitor transfusions. A district hospital physician states, ‘Adequate number of nurses and specialists must be posted at a high case load facility like ours to manage complicated cases and to ensure monitoring during blood transfusion’ [Participant 16].

At the policy level, providers describe the following solutions: rigorous certification of blood banks in Bihar, establishment of protocols for communication between district hospitals and blood banks, hospital responsibility for procuring replacement donation, and non-governmental organization sponsored blood donation camps and blood donor pools. For obstetric indications, a district hospital physician advocated that ‘blood must be issued without replacement. As a policy, blood is issued without replacement only to HIV and thalassemia patients’ [Participant 13]. One physician believed that UDBT should be regulated and allowed – ‘And finally I think this unbanked thing – I think most of the rural areas across the country, even though it’s not legal this is how it’s being done. So maybe if we could have certain standard operating procedures for that… a strict SOP would be in place that only in these criteria can it be used’ [Participant 3].

## Discussion

Lack of timely access to banked blood is a significant and preventable cause of obstetric and surgical mortality in low-resource settings worldwide [1–5]. The LCoGS calls for global access to a safe and affordable blood supply by 2030 [1]. We describe elements of challenges pertaining to achieving this goal in one of the world’s lowest-resource health settings – the Indian state of Bihar, whose population is equivalent to 13% of that of Europe [13]. The challenges we describe correlate with data from low-resource settings in other parts of the world, particularly in relation to perceptions surrounding blood donation and transfusion, availability of banked blood, and adequacy of workforce and infrastructure capacity [11,20,21]. While our results’ recount of these challenges is not entirely new, the potential solutions identified by our study participants – those on the front lines of obstetric and surgical care delivery – are innovative and meaningful. Since the 2015 publication of the LCoGS Report, global surgery efforts have focused on integrating surgical care into national public health planning through data collection and policy enumeration, with an emphasis on local stakeholder involvement and locally derived solutions [22–25]. However, in the arena of blood, this local data are lacking. Researchers agree that the design and implementation of contextually appropriate, locally driven solutions is a priority area to improve blood transfusion systems in low-resource settings [11,26].

International and Indian literature describes misconceptions about blood donation and transfusion as a common barrier to maintaining an adequate stock of blood, and as one of myriad reasons why many LMIC rely heavily on replacement or paid blood donation [27–30] Our findings highlight that in a context where replacement donation is required to maintain blood stores, these misbelieves have dangerous consequences for already-vulnerable patients and physicians. The evidence base for pragmatic, culturally sensitive approaches to donor recruitment and retention is building [11,31–33]. Published strategies have increased voluntary non-remunerated donation rates in African countries by targeting youth with incentivized donation goals and using creative venues such as radio stations for blood donation [34,35]. The providers included in our study advocate for interventions to counter misconceptions about blood donation, such as creating informational videos, providing in-person seminars, and inviting past donors to share about their experience with those considering donation. Our participants also enumerate a role for non-governmental organizations in organizing mass blood donation camps or educating, motivating, and organizing a pool of pre-screened, voluntary donors to be called upon when needed. Taken together, these proposed solutions highlight a key area for local- and national-level intervention, civil society engagement, and continued academic research to use principles of behavior change science in creating, testing, and adopting community education and mobilization strategies.

Providers describe a lack of adequately trained blood bank and nursing workforce and of adequately supplied laboratories, blood banks, and blood storage centers as barriers to timely blood transfusion. A robust body of research has assessed surgical capacity in LMIC public and private hospital systems and found that deficits in infrastructure, equipment, supplies, and trained personnel limit surgical care delivery [3,36–38]. Our findings suggest that these health-system deficiencies dramatically affect providers’ decisions to transfuse or refer patients, and in turn impact obstetric patients’ surgical care. Providers recommend immediate, low-cost solutions – such as stocking labor rooms with units of O negative blood – to mitigate the impact of limited infrastructure and personnel on patient care while longer-term capacity-building interventions are implemented. Thechorus of Indian surgeons and activists have proposed another solution – legalization of UDBT, in which physicians directly transfuse blood from a screened donor to the patient [7,8,39]. Both of these solutions would be illegal under current Indian law [7,40]. Thus while infrastructure and workforce are two of five components of a national surgical plan framework, national level stakeholders must also recognize the urgent need for immediate workarounds and amended laws [41]. Further, as argued previously, academic global surgery research should shift from characterization of infrastructure and workforce deficits to rigorous study of proposed solutions, such as UDBT, to inform implementation [36].

In Bihar, as in other states of India and many LMIC, a poorly coordinated national- and state-level blood transfusion system exacerbates the lack of functional infrastructure and trained workforce [11,42,43]. Our participants illustrate that perverse local policies and absent local-level coordination within this fragmented system cause life-threatening delays in procuring blood, especially for district hospital patients with limited literacy and financial resources. In addition to targeted changes to such policies, state and regional support for enhanced coordination is an absolute necessity. Transparency in supplies and blood availability would be a reasonable first start. The Indian state of Maharashtra is a leader in this regard – providing an online portal to locate a unit of a specific blood type, check blood bank stock, or download donation statistics – and is worthy of emulation [44].

This study is limited by several characteristics of the respondent sample. Snowball sampling created an unequal distribution of gender and practice setting. Despite standardization with an interview manual, the different backgrounds of the two interviewers may have introduced bias. Finally, by interviewing providers, we present an end-user perspective on a system-level issue that would benefit from patient, administrator, and allied health professional voices for a complete picture. In addition, interviews were not transcribed from a recorder and five were conducted over the phone. Thus, important nuance from the interview transcripts and non-verbal cues may not be reflected in the data. Despite these limitations, we provide a limited but rigorous assessment of the effect of a known surgical system barrier on frontline providers in a unique context.

## Conclusions

In summary, meeting the priority of safe access to blood in Bihar requires a multifactorial approach to increase blood donation through education and organization, mitigate limited health system workforce and infrastructure through immediate, organized workarounds, and improve local-level coordination through state support and policy change. Ultimately, a structured approach with involvement from multiple stakeholders must be undertaken to develop and implement solutions, and can build on previously initiated national surgical planning efforts [22,45,46]. The academic global surgery community can and should continue to contribute through high-quality research to guide and monitor implementation efforts.

## Paper context

Lack of timely, safe, affordable access to blood in many low-resource settings across the globe limits access to surgical care, leading to unnecessary patient deaths. This paper adds rigorous data from front-line providers’ experiences in an Indian state with a known deficit of blood. The innovative solutions identified by these providers meaningfully add local input to an ongoing multi-stakeholder, global process of integrating surgical care into national public health planning.
